# *E. tapos* Yoghurt—A View from Nutritional Composition and Toxicological Evaluation

**DOI:** 10.3390/foods11131903

**Published:** 2022-06-27

**Authors:** Ruth Naomi, Rusydatul Nabila Mahmad Rusli, Santhra Segaran Balan, Fezah Othman, Azmiza Syawani Jasni, Siti Hadizah Jumidil, Hasnah Bahari, Muhammad Dain Yazid

**Affiliations:** 1 Department of Human Anatomy, Faculty of Medicine and Health Sciences, Universiti Putra Malaysia, Serdang 43400, Malaysia; ruthmanuel2104@gmail.com (R.N.); rusydatulnabila17@gmail.com (R.N.M.R.); santhra@msu.edu.my (S.S.B.); hadizah_jumidil@upm.edu.my (S.H.J.); 2 Faculty of Health and Life Sciences, Management and Science University, Shah Alam 40100, Malaysia; 3 Department of Biomedical Sciences, Faculty of Medicine and Health Sciences, Universiti Putra Malaysia, Serdang 43400, Malaysia; fezah@upm.edu.my; 4 Department of Medical Microbiology and Parasitology, Faculty of Medicine and Health Sciences, Universiti Putra Malaysia, Serdang 43400, Malaysia; azmiza@upm.edu.my; 5 Centre for Tissue Engineering and Regenerative Medicine, Faculty of Medicine, Universiti Kebangsaan Malaysia, Kuala Lumpur 56000, Malaysia

**Keywords:** *E. tapos* yoghurt, nutritional property, safety, animal model, toxicity study

## Abstract

*Elateriospermum tapos* (*E. tapos*) is a natural tropical plant that possess a wide range of health benefits. Recent discovery proves that *E. tapos* extract is able to reduce weight, increase cognitive performance, and ameliorate anxiety and stress hormone. However, this extraction has not been incorporated into yoghurt, and no toxicity studies have been done previously to prove its safety. Thus, this study was aimed to formulate the ethanolic extracted *E. tapos* into yoghurt and access the toxicological effects on rodents. Forty female Sprague Dawley (SD) rats were used in this study and force fed with either one of the following doses of 250, 500, 1000, or 2000 mg/kg, while the control group received normal saline. The nutritional analysis result showed that the newly formulated yoghurt comprised 328 kJ of energy per 100 mL of servings, 3.6 g of fats, 8.2 g of carbohydrates, 2.7 g of total protein, and 1.2 g of fibre. The peak intensity of *Lactobacillus* species was observed at 1.6 × 10^5^ CFU/g with a titratable acidity as lactic acid of 0.432 CFU/g, indicating the ability of the formulated yoghurt in stimulating the growth of *Lactobacilli*. In the experimental study, the *E. tapos* yoghurt in a single dose (2000 mg/kg) did not show any treatment related to toxicity in any of the rats observed in an additional 14 days. There were no changes in body weight, food and water intake, plasma biochemistry (ALT, AST, ALP, and creatinine), haematological products, and organ weights of the treated groups compared to the subacute control groups. Histological examination of all organs including liver, heart, and kidney were comparable to the control groups. In toto, oral consumptions of *E. tapos* yoghurt did not induce any adverse effects on rodents.

## 1. Introduction

Yoghurt is also known as a concentrated form of milk, made from selective bacterial fermentation [1]. Over the past years, yoghurt has gained the attention of researchers around the globe. For instance, Greek yoghurt was widely known for its thin structure before the introduction of high-protein yoghurt due to its global marketing potentials. However, formulation of thick, creamy yoghurts have suppressed the market of Greek yoghurt [2]. This is because scientists claim that thick and creamy yoghurts were said to be the primary option for consumers compared to the thin and watery type of yoghurt [3]. The consumers’ stipulations for yoghurt will tend to increase in the future due to its potent beneficial effects and due to its inclusion of fewer additives [4] and its nutritional value [1]. Regardless of age group, yoghurt possesses the ability to increase amino acids content of a protein and may enhance the synthesize of proteins in the muscles [5]. Yoghurt tends to contribute to faster satiety and is thereby advantageous for those in calorie-restricted diets [6]. The bacterial strain in yoghurt makes it an ideal source of anti-obesity agents, and studies have proven that these bacterial cultures are able to tolerate the acidity in the gut and survive in the intestinal tract [7]. Thus, lipolysis, amelioration of cognitive function, and reduction in the absorption of cholesterol are commonly documented features in those who consume yoghurts [8]. Moreover, bacterial fermentation processes during yoghurt preparation transform the lactose in milk to lactic acids, which further gives the option for lactose-intolerant subjects to consume yoghurt without any allergic reactions [9]. In these, integration of natural medicinal plants into yoghurt is a new insight in biomedical research.

*Elateriospermum tapos* (*E. tapos*) is a monotypic plant, a tropical canopy found in the deep forest of Southeast Asia, commonly in Malaysia, Thailand, Sumatera, and Java [10], and has raised our interest due to its beneficial composition. *E. tapos* belongs to the family Euphorbiaceae and is known as “*buah perah*” locally [11]. Numerous bioactive compounds have been identified from *E. tapos* extract, including flavonoids, tannins, alkaloids [12], linolenic acids, polyunsaturated fats [10], phenols, saponins, sterols, proteins, and iodine [13]. *E. tapos* extract has been proven to enhance antioxidant activity, inhibit pancreatic lipase [11], ameliorate stress hormone, and act as an anti-obesity agent [10]. Due to its potential health effects, *E. tapos* recently has attracted researchers’ attention to pursue further investigations in animal studies. According to the recent scientific findings, yoghurt is usually flavoured via natural plants or fruits to provide colour and bioactive compounds [1]. As such, mixture of *E. tapos* extraction, a local fruit, into yoghurt gives rise to a whole new formulation of yoghurt product. However, there is not any previous study that has introduced or evaluated the nutritional composition of *E. tapos* yoghurt or assessed its toxicity. Hence, this article discusses the novel yoghurt formulation supplemented with ethanolic extraction of *E. tapos* and its nutritional composition and toxicological evaluation on SD rats.

## 2. Results

### 2.1. Nutritional Composition

The formulated *E. tapos* yoghurt appears white, creamy, sticky, consistent, smooth, firmly textured, and with typical yoghurt-like smell. It has a pH of 5.1 (slightly acidic) at 25 °C with a titratable acidity as lactic acid 0.4%. The nutritional compositions of the *E. tapos* yoghurt are as tabulated in Table 1.

### 2.2. Acute Toxicity Study

#### 2.2.1. Clinical Observation

The administration of *E. tapos* yoghurt at 2000 mg/kg did not result in mortality in any of the treated rats. No abnormalities or changes in behavioural pattern were recorded. The observation on gross pathology of acute *E. tapos* 2000 mg/kg (AT 2000) on liver, heart, and kidney showed no changes in colour, shape, and size compared to the acute control (AC) group. The scoring sheet documented shows 100% normal texture.

#### 2.2.2. Body Weight Changes

The changes in body weight between AC group and AT 2000 group are shown in Table 2. There were gradual increase in body weight of both AC and AT 2000 groups. There were no significant difference (*p* > 0.05) of the AT 2000 group compared to the AC group from day 0, week 1, and week 2.

#### 2.2.3. Total Food and Water Consumption

Table 3 shows the data for food consumption and water intake in acute toxicity study. There were no significant differences (*p* > 0.05) of the AT 2000 group compared to the AC group in total food consumption, calorie intake, food efficiency ratio (FER), and total water intake.

#### 2.2.4. Relative Organ Weight (ROW)

The ROW for AC and AT 2000 are shown in Table 4. There were no significant difference (*p* > 0.05) of the AT 2000 group compared to the AC group in the organ weight (g) and standardized by body weight (%). The correct value for the relative organ weight of the spleen is 0.001, as accurately reflected in the table below. An unintended formatting discrepancy occurred during the preparation of the manuscript, where three decimal points were included, rendering the value as 0.00. To maintain consistency with other tables in the manuscript, we will correct the formatting to present the value as 0.00 in accordance with the established convention.

#### 2.2.5. Haematological Analysis

The data for haematological analysis are presented in Table 5 below. The supplementation of *E. tapos* 2000 mg/kg yoghurt caused a significant decrease (*p* < 0.05) in mean corpuscular volume (MCV) compared to the AC group. However, no significant difference (*p* > 0.05) was observed in other measured parameters between the AT 2000 and AC groups.

#### 2.2.6. Blood Biochemistry Analysis

The blood biochemistry analysis is presented in Table 6. A significant increase (*p* < 0.05) in albumin and albumin globulin ratio is shown in the AT 2000 group compared to the AC group. Contrarily, a significant decrease (*p* < 0.05) in urea, globulin, ALP, and AST concentration was shown in the AT 2000 group compared to AC group. However, in other blood biochemistry parameters measured, there were no significant difference (*p* > 0.05) between the AT 2000 and AC group.

#### 2.2.7. Histopathological Analysis

**Figure 1:**
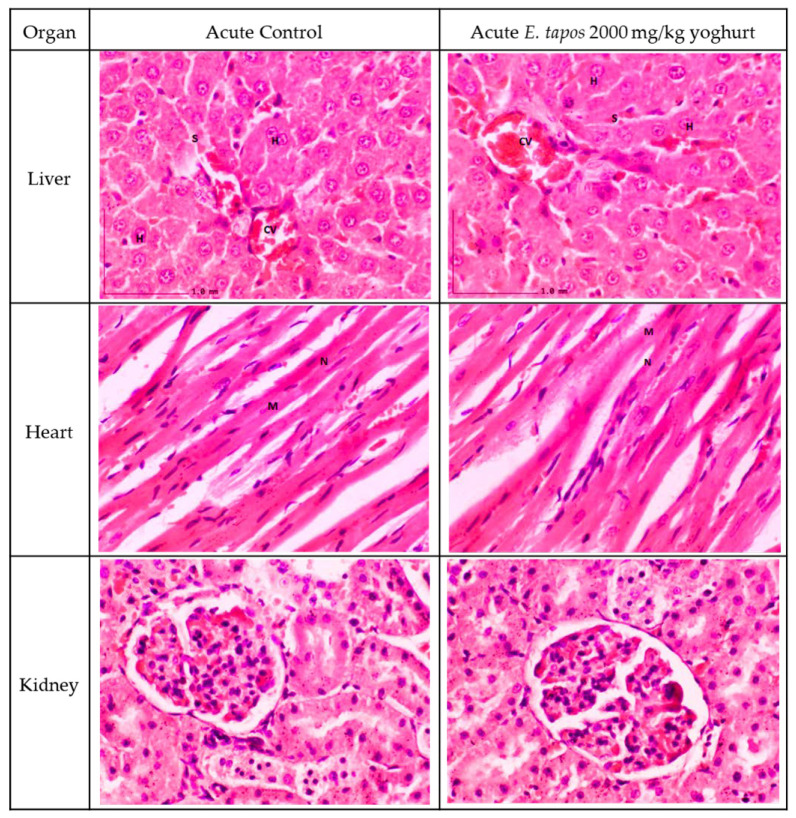
Histology section of acute toxicity study. The liver section shows standard strands of hepatocytes (H), sinusoids (S), and central vein (CV). It shows 0% of biopsied hepatocytes affected. The liver grading score was recorded as 0 due to absence of steatosis, lobular inflammation, and hepatocyte ballooning. The heart histological section shows normal heart architecture, and no inflammation was observed. Myocardium (M) and nucleus (N). The kidney histology showed no abnormal lesion or tubular dilation. Renal corpuscle appeared normal. No abnormalities were observed in kidney histology section.

The histological section for liver, kidney, and heart are illustrated in Figure 1. The histological examination shows no pathological abnormalities and the histological section of kidney, liver, and heart for AT 2000 when compared to the AC group. The kidney for control and treated groups showed normal proximal and distal tubule. The mesangial cells in the glomerulus were observed. Bowman space between the visceral and parietal layer and the cup-like sac surrounding the glomerulus were observed. The liver in the treated groups showed normal hepatic architecture; no hepatocytes ballooning or hepatocyte degeneration, inflammation, or other pathological abnormalities were observed. The transverse striation of cardiac muscle cells, a central nucleus, and epicardium with the presence of mesothelial pavement cells were observed in heart tissue.

### 2.3. Subacute Toxicity Study (Repeated Dose)

#### 2.3.1. Clinical Observation

The administration of *E. tapos* yoghurt at various concentrations of 250, 500, and 1000 mg/kg for 28 days and satellite dose of 1000 mg/kg for 28 days followed by absence of treatment for 14 days did not exhibit any form of physical abnormalities nor mortality. There were no abnormalities concerning the hair coat, eye colour, salivation, diarrhoea, touch response, tail pinch, and grip strength. Any clinical signs of locomotor dysfunction, tremors, or convulsions were absent, and all animals survived until necropsy. Gross observations did not reveal any treatment-related changes in treated animals.

#### 2.3.2. Body Weight Changes

The changes in body weight were recorded weekly for 28 days for repeated dose, while 42 consecutive days were recorded for the subacute satellite group as shown in Table 7. In subacute toxicity, no significant changes (*p* > 0.05) in weekly body weight changes were shown in subacute *E. tapos* 250 mg/kg of yoghurt (ST 250)-, subacute *E. tapos* 500 mg/kg of yoghurt (ST 500)-, and subacute *E. tapos* 1000 mg/kg of yoghurt (ST 1000)-treated group compared to subacute control (SC) in day 0 and week 2, 3, and 4. There were significant changes (*p* < 0.05) between ST 250-treated group compared to SC in week 1, while there were no significant changes (*p* > 0.05) recorded between ST 500 and ST 1000 compared to SC in week 1. As in the satellite group, no significant difference (*p* > 0.05) was shown in weekly body weight changes between the treated group of subacute satellite *E. tapos* 1000 mg/kg of yoghurt (SST 1000) and subacute satellite control (SSC) group.

#### 2.3.3. Total Food and Water Consumption

Table 8 represents total food consumption, caloric intake, FER, and total water intake for subacute toxicity study of SC, ST 250, ST 500, and ST 1000 for 28 days with repeated dose and SSC and SST 1000 for 42 consecutive days. No significant difference (*p* > 0.05) were shown for FER, calorie intake, and total food and water consumption in ST 250-, ST 500-, and ST 1000-treated groups compared to SC. Meanwhile, in the satellite group for subacute toxicity, no significant difference (*p* > 0.05) were observed in FER, calorie intake, total food and water consumption in SST 1000-treated group compared to SSC group.

#### 2.3.4. Relative Organ Weight (ROW)

Table 9 shows the data for organs weight in rats for subacute toxicity study of SC, ST 250, ST 500, and ST 1000 for 28 days with repeated dose and SSC and SST 1000 for 42 consecutive days. There were no abnormalities observed for liver, spleen, RpWAT, visceral fat, inguinal fat, and heart. In subacute toxicity, no significant difference (*p* > 0.05) were recorded in ROW and in a percentage per body weight of liver, spleen, kidney, RpWAT, visceral, inguinal, lung, and heart in rats supplemented with ST 250, ST 500, and ST 1000 *E. tapos* yoghurt compared to SC group. The satellite group for subacute toxicity shows a significant increase (*p* < 0.05) for kidney in ROW of SST 1000 compared to SSC. However, there were no significant difference (*p* > 0.05) in ROW and organ percentage per body weight in group SST 1000 compared with the SSC group for liver, spleen, RpWAT, visceral, inguinal, lung, and heart.

#### 2.3.5. Haematological Analysis

The effects of daily administration of *E. tapos* yoghurt at various doses of SC, ST 250, ST 500, and ST 1000 for 28 days with repeated dose and SSC and SST 1000 for 42 consecutive days in haematological parameters are shown in Table 10. There were significant decrease (*p* < 0.05) in WBC of ST 500 compared to SC and significant decrease (*p* < 0.05) of lymphocytes of ST 500 and ST 1000 compared to SC. However, there were no significant difference (*p* > 0.05) on Hb (g/dL) RBC, RDW, PCV, MCV, MCH, MCHC, neutrophils, eosinophils, basophils, and platelets in ST 250, ST 500 and ST 1000 groups compared to SC group. However, in the satellite group for subacute toxicity, a significant decrease (*p* < 0.05) for WBC, lymphocytes, and eosinophils was observed in the SST 1000 group compared to the SSC group. However, there was not any significant difference observed in other haematological parameters in the SST 1000 group compared to SSC group.

#### 2.3.6. Blood Biochemistry Analysis

The blood biochemistry data on the effect of *E. tapos* yoghurt on subacute toxicity study for SC, ST 250, ST 500, and ST 1000 for 28 days with repeated dose and SSC and SST 1000 for 42 consecutive days are presented in Table 11 below. There was significant decrease (*p* < 0.05) in urea and creatinine of the ST 1000 compared to the SC group. Similarly, there was significant increase (*p* < 0.05) in ALP of ST 500 and ST 1000 compared to the SC group. However, there were no significant changes (*p* > 0.05) in sodium, potassium, chloride, total protein, albumin, globulin, albumin–globulin ratio, AST, ALT, and gama-glutamyl transferase, while in the satellite group for subacute toxicity, there were significant decrease (*p* < 0.05) in urea in SST 1000 group compared to the SSC group. However, there was no significant difference observed in other blood biochemical parameters in SST 1000 group compared to SSC group.

**Table 8:** Total food consumption, caloric intake, FER, and total water consumption in the subacute toxicity study. Values are expressed as mean ± SEM.

	Groups for Subacute Toxicity ^1^				Satellite Group for Subacute Toxicity ^2^	
	SC	ST 250	ST 500	ST 1000	SSC	SST 1000
Total food consumption (g)	456.42 ± 6.44	454.84 ± 30.80	455.89 ± 23.87	443.78 ± 19.18	825.78 ± 14.57	829.33 ± 9.98
Calorie intake (KJ)	5842.22 ± 82.43	5821.91 ± 394.24	5835.35 ± 305.54	5680.34 ± 245.50	10,569.96 ± 186.52	10,615.36 ± 127.68
FER (%)	15.73 ± 1.57	17.59 ± 0.16	16.78 ± 0.06	16.47 ± 3.33	12.74 ± 0.70	13.80 ± 1.20
Total water intake (mL)	956.67 ± 0.00	927.50 ± 5.83	933.33 ± 70.00	921.67 ± 11.67	1598.33 ± 58.33	1615.83 ± 122.50

^1^ Groups for subacute Toxicity. ^2^ Satellite groups for subacute toxicity.

**Table 9:** Relative organ weight of rats treated with *E. tapos* yoghurt in the subacute toxicity study. Values are expressed as mean ± SEM. (*) indicates a significant difference at *p* < 0.05.

Organs	Groups for Subacute Toxicity ^1^				Satellite Group for Subacute Toxicity ^2^	
	SC	ST 250	ST 500	ST 1000	SSC	SST 1000
Relative organ weight (g)						
Liver	8.40 ± 0.65	9.33 ± 0.58	9.04 ± 0.43	8.46 ± 0.56	8.98 ± 0.93	9.79 ± 0.66
Spleen	0.59 ± 0.05	0.51 ± 0.05	0.56 ± 0.07	0.78 ± 0.32	0.45 ± 0.02	0.54 ± 0.07
Kidney	1.61 ± 0.10	1.78 ± 0.09	1.72 ± 0.09	2.03 ± 0.33	1.71 ± 0.07 *	2.03 ± 0.09 *
RpWAT	1.73 ± 0.28	1.58 ± 0.24	1.46 ± 0.20	1.43 ± 0.21	1.81 ± 0.23	1.92 ± 0.26
Visceral fat	3.47 ± 0.51	3.12 ± 0.61	3.32 ± 0.71	2.47 ± 0.48	3.93 ± 0.45	2.94 ± 0.76
Inguinal fat	1.22 ± 0.25	1.15 ± 0.18	1.44 ± 0.31	1.62 ± 0.38	1.25 ± 0.39	1.47 ± 0.28
Lung	1.81 ± 0.07	2.05 ± 0.10	1.85 ± 0.05	1.84 ± 0.19	1.89 ± 0.11	2.08 ± 0.13
Heart	0.93 ± 0.04	1.11 ± 0.16	0.90 ± 0.04	0.93 ± 0.02	0.92 ± 0.04	1.09 ± 0.08
Percentage per body weight (%)						
Liver	3.48 ± 0.35	3.64 ± 0.21	3.67 ± 0.21	3.45 ± 0.24	3.46 ± 0.41	3.58 ± 0.26
Spleen	0.25 ± 0.03	0.20 ± 0.02	0.22 ± 0.03	0.32 ± 0.13	0.17 ± 0.01	0.20 ± 0.03
Kidney	0.67 ± 0.05	0.70 ± 0.04	0.70 ± 0.04	0.83 ± 0.14	0.66 ± 0.04	0.74 ± 0.04
RpWAT	0.70 ± 0.11	0.62 ± 0.09	0.59 ± 0.09	0.58 ± 0.08	0.70 ± 0.10	0.70 ± 0.09
Visceral fat	1.44 ± 0.23	1.22 ± 0.24	1.34 ± 0.28	1.01 ± 0.20	1.51 ± 0.19	1.08 ± 0.28
Inguinal fat	0.49 ± 0.09	0.45 ± 0.07	0.60 ± 0.14	0.66 ± 0.15	0.49 ± 0.16	0.55 ± 0.11
Lung	0.74 ± 0.04	0.80 ± 0.04	0.75 ± 0.04	0.75 ± 0.08	0.72 ± 0.05	0.76 ± 0.05
Heart	0.38 ± 0.03	0.43 ± 0.06	0.36 ± 0.02	0.38 ± 0.01	0.35 ± 0.01	0.40 ± 0.03

^1^ Groups for subacute Toxicity. ^2^ Satellite groups for subacute toxicity.

**Table 10:** The haematological effects of the different treatment dose of *E. tapos* yoghurt on female SD rats in the subacute toxicity study. Values are expressed as mean ± SEM. (*) indicates a significant difference at *p* < 0.05.

	Groups for Subacute Toxicity ^1^				Satellite Group for Subacute Toxicity ^2^	
Parameters	SC	ST 250	ST 500	ST 1000	SSC	SST 1000
Hb (g/dL)	15.77 ± 0.45	14.85 ± 0.21	15.07 ± 0.21	15.37 ± 0.32	14.85 ± 0.32	15.08 ± 0.21
RBC (×10^−12^/L)	8.49 ± 0.26	8.15 ± 0.14	8.25 ± 0.17	8.39 ± 0.26	8.37 ± 0.22	8.38 ± 0.17
RDW (%)	14.02 ± 0.43	13.27 ± 0.26	13.78 ± 0.19	14.67 ± 0.63	15.02 ± 0.29	14.90 ± 0.52
PCV (%)	47.50 ± 1.67	45.00 ± 0.73	45.33 ± 0.71	45.00 ± 1.03	45.50 ± 0.99	46.17 ± 0.48
MCV (fL)	56.00 ± 0.45	55.33 ± 0.76	54.83 ± 0.54	53.83 ± 0.83	54.33 ± 0.42	55.00 ± 0.52
MCH (pg)	18.67 ± 0.21	18.33 ± 0.33	18.33 ± 0.33	18.50 ± 0.34	18.00 ± 0.00	18.17 ± 0.17
MCHC (g/dL)	33.33 ± 0.33	33.17 ± 0.31	33.50 ± 0.43	34.17 ± 0.48	32.83 ± 0.17	32.83 ± 0.31
WBC (×10^−9^/L)	8.98 ± 1.31 *	7.72 ± 0.69	5.35 ± 0.73 *	5.47 ± 0.61	9.70 ± 0.86 *	5.62 ± 1.14 *
Neutrophils (×10^−9^/L)	2.22 ± 0.65	1.48 ± 0.20	1.15 ± 0.11	1.55 ± 0.38	1.47 ± 0.18	1.05 ± 0.17
Lymphocytes (×10^−9^/L)	6.25 ± 0.77 *	5.73 ± 0.49	3.82 ± 0.59 *	3.57 ± 0.34 *	7.85 ± 0.73 *	4.23 ± 0.93 *
Monocytes (×10^−9^/L)	0.20 ± 0.04	0.18 ± 0.05	0.15 ± 0.05	0.20 ± 0.09	0.10 ± 0.00	0.22 ± 0.16
Eosinophils (×10^−9^/L)	0.30 ± 0.05	0.25 ± 0.01	0.23 ± 0.07	0.13 ± 0.01	0.22 ± 0.04 *	0.11 ± 0.03 *
Basophils (×10^−9^/L)	0.00 ± 0.00	0.02 ± 0.02	0.00 ± 0.00	0.00 ± 0.00	0.02 ± 0.02	0.00 ± 0.00
Platelet (×10^−9^/L)	777.33 ± 162.16	499.17 ± 127.46	758.00 ± 204.29	459.50 ± 206.47	1028.17 ± 84.56	783.83 ± 172.72

^1^ Groups for subacute Toxicity. ^2^ Satellite groups for subacute toxicity.

**Table 11:** The effects of *E. tapos* yoghurt on biochemistry analysis in the subacute toxicity studies. Values are expressed as mean ± SEM. (*) indicates a significant difference at *p* < 0.05.

	Groups for Subacute Toxicity ^1^				Satellite Group for Subacute Toxicity ^2^	
Parameters	SC	ST 250	ST 500	ST 1000	SSC	SST 1000
Sodium (mmol/L)	137.17 ± 0.40	138.50 ± 0.76	139.00 ± 0.52	137.67 ± 0.33	138.00 ± 0.52	138.50 ± 0.43
Potassium (mmol/L)	8.53 ± 0.16	7.98 ± 0.26	8.57 ± 0.20	8.50 ± 0.29	8.85 ± 0.24	8.30 ± 0.28
Chloride (mmol/L)	101.83 ± 0.48	101.00 ± 1.03	101.50 ± 1.20	102.83 ± 0.48	100.00 ± 0.93	102.17 ± 0.31
Urea (mmol/L)	8.13 ± 0.48 *	8.08 ± 0.45 *	7.85 ± 0.55	6.23 ± 0.30 *	7.65 ± 0.40 *	6.38 ± 0.19 *
Creatinine (mmol/L)	41.17 ± 1.83 *	38.00 ± 1.06	36.50 ± 2.11	31.50 ± 1.23 *	34.50 ± 5.05	37.17 ± 1.14
Total Protein (g/L)	66.83 ± 1.49	66.33 ± 0.84	70.00 ± 0.63	66.50 ± 1.09	74.17 ± 1.58	70.83 ± 1.38
Albumin (g/L)	38.67 ± 1.02	39.67 ± 0.92	41.67 ± 1.05	39.67 ± 0.61	42.33 ± 0.88	41.17 ± 0.98
Globulin (g/L)	28.17 ± 0.79	26.67 ± 0.42	28.33 ± 0.67	26.83 ± 0.75	31.83 ± 1.01	29.67 ± 0.61
Albumin–Globulin ratio	1.38 ± 0.04	1.49 ± 0.05	1.48 ± 0.07	1.48 ± 0.04	1.34 ± 0.04	1.39 ± 0.03
ALP (U/L)	86.00 ± 3.57 *	72.00 ± 2.96 *	84.33 ± 6.72 *	113.00 ± 5.59 *	62.00 ± 7.11	66.83 ± 4.87
AST (U/L)	104.67 ± 5.61	99.17 ± 9.42	120.50 ± 18.71	114.83 ± 10.58	92.67 ± 15.57	84.00 ± 7.60
ALT (U/L)	33.67 ± 4.01	36.83 ± 3.16	41.83 ± 3.89	35.50 ± 1.88	47.83 ± 8.89	34.50 ± 2.93
Gama-Glutamyl Transferase (U/L)	10.83 ± 1.01	11.67 ± 0.61	11.50 ± 0.56	10.83 ± 0.91	13.00 ± 0.00	13.00 ± 0.00

^1^ Groups for subacute Toxicity. ^2^ Satellite groups for subacute toxicity.

#### 2.3.7. Histopathological Analysis

**Figure 2:**
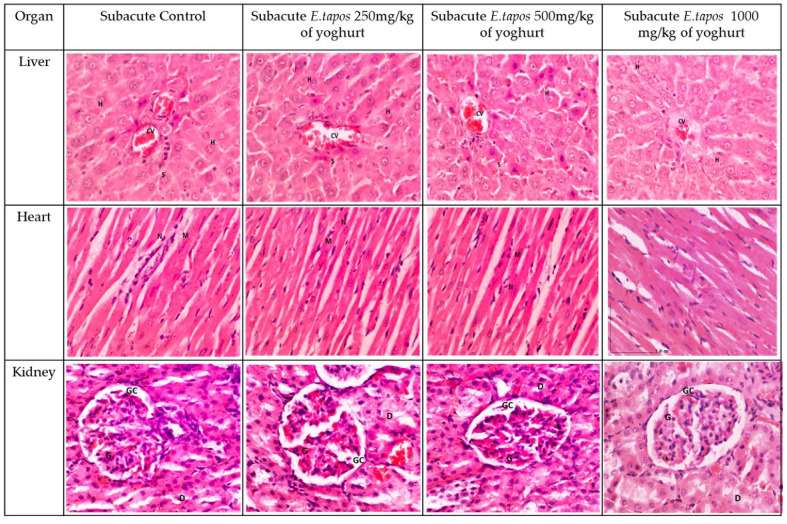
The histology section for subacute studies. The liver section shows standard strands of hepatocytes (H), sinusoids (S), and central vein (CV). It shows 0% of biopsied hepatocytes affected. The liver grading scores were documented as 0 due to absence of steatosis, lobular inflammation, and hepatocyte ballooning. The heart tissue showing normal heart architecture and no inflammation was observed. Myocardium (M) and nucleus (N). Kidney histology showed no lesion or tubular dilation. Renal corpuscle appeared normal. The kidney shows normal architecture of glomerulus (G), glomerulus capsule (GC), distal convoluted tubule (D). No abnormalities observed in the liver, kidney, or heart in subacute toxicity groups.

**Figure 3:**
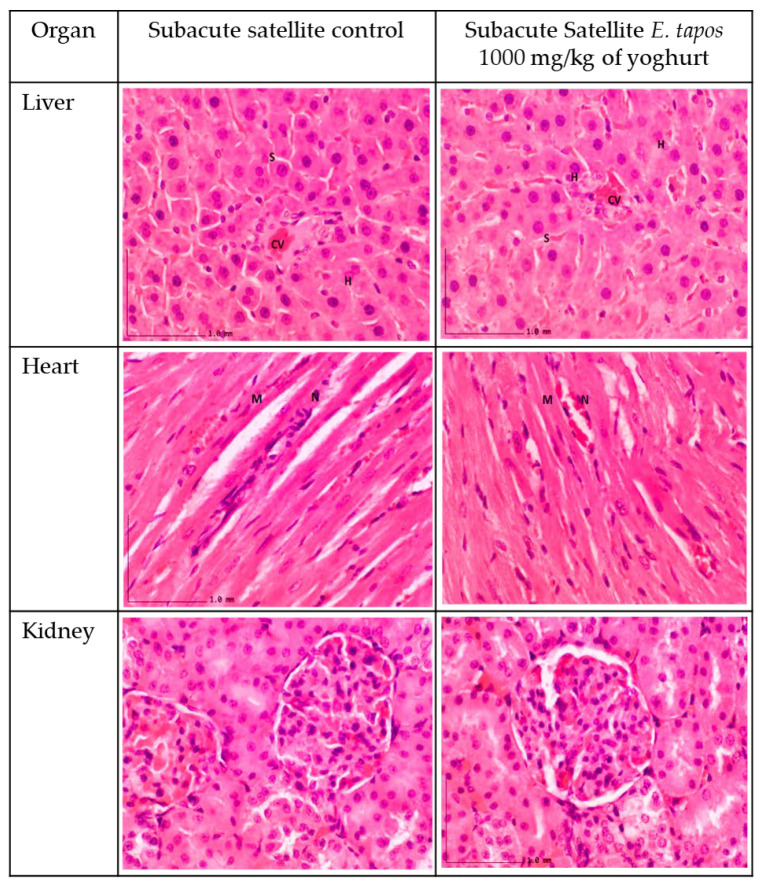
The histology section of satellite groups. The liver section shows standard strands of hepatocytes (H), sinusoids (S), and central vein (CV). It showed 0% of biopsied hepatocytes affected. The liver grading scores were recorded as 0 due to absence of steatosis, lobular inflammation, and hepatocyte ballooning. The heart tissue shows normal architecture, and no inflammation was seen. Myocardium (M) and nucleus (N). Kidney histology show no lesion nor tubular dilation with a normal structure of renal corpuscle.

The histological observation on the liver, kidney, and heart section for subacute studies are shown in Figure 2 and Figure 3, respectively. The histopathological studies of the liver sections in the SC group showed a normal appearance of CV and hepatic sinusoids lined by endothelial cells (EC) with normal radiating hepatocytes. The rats supplemented with ST 250, ST 500, and ST 1000 showed a similar CV and sinusoids lined with EC with normal radiating hepatocytes as observed in the SC group. The SST 1000 showed a similar liver architecture structure as the SSC group, with no inflammation or congestion of WBC observed in group. The transverse striation of cardiac muscle cells, a central nucleus, and epicardium with the presence of mesothelial pavement cells were observed in heart tissue.

## 3. Discussion

This study is divided into two phases. In the first phase, also in vitro, the formulated *E. tapos* yoghurt was analysed for its nutritional composition. According to the cut-off point for nutritional composition indicated by European Union (EU) regulations, the formulated *E. tapos* yoghurt can be classified as a low-sugar and -protein yoghurt. This is due to its composition, which is <5 g sugar content per 100 g of *E. tapos* yoghurt and 2.6 g of protein content per 100 g of *E. tapos* yoghurt servings [14]. According to EU guidelines, dairy products such as yoghurt must contain at least 2.7 g/100 g servings for total protein and a maximum of 5 g/100 g of servings for sugars, while for fat should be <3 g/100 g per serving. However, for fat content, the *E. tapos* yoghurt shows a median threshold with 3.6 g per 100 g serving [14]. It contains an appreciable amount of total dietary fibre at 1.2%, providing 9.6 kJ, which enhances satiety effects and reduces energy intake [15]. It provides up to 33 kJ of calorie from digestible carbohydrates [16].

In the second phase, the formulated *E. tapos* yoghurt was tested on female SD rats for safety analysis. The toxicity study was evaluated through acute and subacute toxicity studies (repeated dose). In acute toxicity study, AT 2000, at a single dose of 2000 mg/kg per body weight did not cause any mortality, behavioural abnormalities, postural changes, or drastic body weight changes. However, slight reduction in bodyweight was seen in ST 250 in the first week. Even so, the weight gain and calorie intake were comparable between the ST 250 and the control group during the rest of the experimental period. No weight loss was documented after the first week. Other groups in the subacute and acute study did not reveal any treatment dependent body weight changes or weekly food intake.

The kidney and liver are the most vital organs to rule for toxicity in both animals and humans. The gross weight measurement of kidney could indicate some sort of serious adverse effects. For instance, as noticed in this study (SST 1000 group), the increase in kidney mass could be due to hydronephrosis or nephrotoxicity, as reviewed by previous literature [17]. However, the contraindication of liver profile test results, such as a decreased level of urea, creatinine, globulin, and ALP and AST in the acute or repeated-dose study, confirms the absence for the possibility of hydronephrosis or nephrotoxicity due to *E. tapos* yoghurt’s consumption.

Meanwhile, the level of urea, ALP, AST, and creatinine are some of the biomarkers for renal functionality [18]. Decreased levels of urea, globulin, and creatinine in serum correlates with severe malnutrition or liver diseases [19], while significantly low levels of serum ALP and AST observed, as in subacute phase, contraindicate the possibility of developing malnutrition or liver disease. This is because low levels of ALP and AST prove the absence of protein malnutrition, as in the case of a low-protein diet, where the levels of ALP and AST will increase according to the severity [20]. Moreover, a low-protein diet also is an indicator for decreased levels of urea in serum [21,22]. The primary reason for this is mainly because urea is the end metabolite of the protein catabolism process by the liver [23], while creatinine is the product of creatinine metabolism in the muscles [24].

In contrast, decreased levels of globulin together with increased levels of albumin are a simple indicator for the immune system maturation process. This is because maternally obtained antibodies tend to degrade within the first 6 weeks of birth, and during this period, albumin synthesize will increase [25]. This is an indicator for normal liver formation, and since the SD rats used in this study were 6-week-olds, this hypothesis is acceptable. Non-significant changes observed in all other liver and kidney profile tests in both acute and subacute study may suggest that *E. tapos* yoghurt does not have adverse effects on kidney and liver.

Haematological analysis in toxicity study shows the possibility of toxic effects induced by test material. From the blood biochemistry, a reduction in WBC, specifically lymphocytes, was recorded in both ST 500 and SST 1000 study, while low levels of eosinophils and MCV were documented in the SST 1000 and AT 2000 groups, respectively. Both lymphocytes and eosinophils are involved in immune defence, and usually a slight reduction in these is not something to be concerned with. However, significant reduction of lymphocytes and eosinophils in the animal model usually relates to chronic stress level, such as chronic renal failure or inflammation [26]. However, normal architecture and absence of inflammation in histopathological evaluation of liver, kidney, and heart in both acute and subacute toxicity study confirms the absence of any form of injury to liver or kidney. Low levels of MCV are a clinical sign either in anaemic condition [27] or psychological stress [28]. Yet, normal levels of HB, RBC, MCHC, and MCH rule out the possibility of anaemic condition in treated rats. Thus, the slight decrease in lymphocytes, eosinophils, and MCV most probably could be incidental instead of due to a treatment-related effect. Non-significant changes observed in all other blood biochemistry parameters in both acute and subacute study may suggest *E. tapos* yoghurt does not have effects on haematological products.

From the histopathological perspective, liver, kidney, and heart are the primary organs to be affected in case of stimulatory effect of toxins. Observation on gross morphology on kidney, liver, and heart did not show any form of abnormal texture, colour, or hypertrophy in both acute and subacute study in comparison to AC, SC, or SSC groups. Moreover, organ weight could be a marker of the pathological condition of the rats. Overall, there were no significant differences in ROW documented for liver, kidney, and heart in both acute and subacute studies in comparison with their respective control groups. Microscopic sectioning and staining of liver showed no evidence of inflammation or necrosis. Instead, a normal lobular arrangement was observed. There were neither manifestations of glomerulosclerosis nor scarring of parenchymal tissue in the renal tissue. In the heart, absence of myocardial necrosis was confirmed in both acute and repeated-dose study, further proving the safety of *E. tapos* yoghurt. In consideration with the data obtained from the nutritional analysis and toxicological evaluation study, it can be concluded that single-dose administration of up to 2000 mg/kg/day is a tolerable dosage.

## 4. Materials and Methods

### 4.1. Collection and Confirmation of Plant Species

The fresh *E. tapos* plant was obtained from Research Centre of Forest Research Institute of Malaysia (FRIM), Pahang. The plant materials were identified and deposited at the Medicinal Plant division of FRIM followed by the separation of *E. tapos* seed. The seed was then sent for confirmation at Herbarium Biodiversity Unit (voucher code: UPM SK 3154/17) at the Institute of Bioscience, Universiti Putra Malaysia.

### 4.2. Ethanol Extraction of E. tapos Seed

The *E. tapos* seed was washed with running tap water to remove any external material. About 500 g of seed was soaked in 2000 mL of 95% ethanol in a 2 L conical flask. The conical flask was wrapped with aluminium foil and was left at room temperature for seven days. On the 7th day, the supernatant was collected and filtered through a Whatman paper N°1 filter paper. The solid residue was then repeatedly extracted 3 times with ethanol. The filtrates from each extraction was combined, and all the solvent was evaporated under reduced pressure using a rotary evaporator to yield the crude ethanolic extract of each *E. tapos* seed [29]. The obtained crude extracts were then mixed with maltodextrin in the ratio of 1:1 [30]. The samples were stirred at room temperature and oven dried overnight at 45 °C. The final extracted powder was then stored at −20 °C until further usage.

### 4.3. Preparation of E. tapos Yoghurt

The formula for yoghurt preparation was adapted and modified from methods described elsewhere [31]. About 100 mL of full cream (Dutch Lady Purefarm UHT) milk was pasteurized at 70–75 °C for 30 min using the microwave and cooled down to 45 °C at room temperature. The probiotic starter culture (0.06% *w*/*w*) consisting of a mixture culture of lactose, milk, *Streptococcus thermophilus APC151*, *Lactobacillus delbrueckii subsp. Bulgaricus ATCC 11842,* and autolysed yeast purchased from New England cheesemaking supply company was added to the cooled milk. This was then further incubated in the yoghurt maker (Pensonic PYM-700) at 40–45 °C for 7 h until the pH dropped to 4.5–4.6. The final product of the yoghurt was then refrigerated at 4 °C overnight. The following day, a stock *E. tapos* yoghurt solution was prepared by dissolving 2 g of *E. tapos* fleshes of powder into 100 mL of yoghurt [31].

### 4.4. Color and Visual Appearance

A colorimeter (Chroma meter CR-400, Minolta, Osaka, Japan, equipped with illuminant/observer D65/2°) was used to study the colour changes in yoghurt during the fermentation process. The result was documented as lightness or darkness and yellowness or blueness, as described elsewhere [32].

### 4.5. Determination of pH at 25 C

The pH values of the formulated yogurt were measured using digital potentiometer (420 Benchtop, Orion Research Inc., Beverly, MA, USA) [1].

### 4.6. Titrable Acidity of Lactic Acid

The titrable acidity of lactic acid was determined according to the method described in AOAC (2000). In this, 0.1 M of NaoH was titrated into 10 mL of yoghurt [33]. The acidity was then expressed as gram of lactic acid/100 g using the formula below:

Total acidity = volume × NaOH factor (mL) × 0.009 × yoghurt volume × 100

### 4.7. Determination of Total Sugar

The refractometric method was used to determine the total sugar content in the yoghurt. In this, about 2 to 3 drops of yoghurt was pipetted into the main prism. The appearance of boundary lines in the refraction field was recorded, and the refractive index was identified [34].

### 4.8. Determination of Fat

Gerber protocol was adapted to determine the fat content. In these, 10 mL of ammonia was added to the 100 mL yoghurt and mixed well. Simultaneously, 10 mL of 1.825 g/L sulfuric acid (H_2_SO_4_) was pipetted into the butyrometer. The mixture of yoghurt was then gently pipetted into the butyrometer, allowing the formation of a thin layer on top of the H_2_SO_4_ acid. Then, 1 mL of 0.815 g/L amyl alcohol was dispensed into the butyrometer, inverted, and was centrifuged for 5 min [35]. After centrifuging, the fat content was determined using the formula below:

Fat percentage = (B − A) × 1.1

A = Reading obtained at the base of fat column;

B = Reading obtained at the crest of the fat column.

### 4.9. Determination of Protein Content

The Kjeldahl method was used to measure the protein content. Formulated yoghurt was placed into Kjeldahl flask and digested with H_2_SO_4_ and catalyst. This was followed by dilution with H_2_O and neutralization with sodium thiosulfate to obtain boric acid solution. Hydrochloric acid was then used to titrate the borate anions, which eventually forms nitrogen [36]. The crude protein was calculated by multiplying with the conversion factor of 6.38 using the formula below:

% N × 6.38 = % protein

### 4.10. Determination of Carbohydrate

Phenol sulfuric acid method was used to determine the carbohydrate content. First, 200 µL of 5% of phenol was added to the 200 µL of yoghurt in a test tube. This was followed by adding 1 mL of concentrated H_2_SO_4_ and vortex. The mixture in the test tube was then incubated in room temperature for 60 min and was read using a spectrophotometer at 490 nm absorbance [37].

### 4.11. Determination of Energy

The energy content of the formulated yoghurt was calculated based on the method JKM F 1205 based on the guidance of Nutrition Labelling and Claims by Food safety and quality Division MOH described in the Malaysian food composition database [38]. The formula used for energy (kcal) calculations are as below:

Energy, kcal/100 g = [Fat] × 9 + [Protein] × 4 + [Carbohydrate] × 4

Energy, kJ/100 g = kcal × 4.184

### 4.12. Determination of Total Dietary Fibre

Dietary fibre content was determined according to the procedure elsewhere [33]. In this, the formulated yoghurt was freeze dried until the powder form was obtained. The powder form of yoghurt was then placed into digestion flask, and 200 mL of 1.25% of H_2_SO_4_ was added. It was boiled for about 30 min with frequent rotation until the samples became completely wet. Thereafter, the samples were filtered with fluted funnel and washed with boiling water. The residue was again washed with 200 mL of 1.25% of H_2_SO_4_ and boiled for 30 min via reflux condenser. Thereafter, the samples were filtered with an asbestos mat, and the residue was again washed with boiling water, followed by 10 mL of alcohol, and dried at 110 °C (W_1_). The residue was then transferred into the muffle furnace at a controlled temperature of 525 to 550 °C, and the ash material was used to obtain the value of W_2_. The loss of weight represents the crude fibre [33].

### 4.13. Survival of Lactic Acid Bacteria

The method was adapted and modified from Vinderola et al., 2003. In this, approximately 25 mL of yoghurt was diluted with 225 mL of peptone H_2_O in a bag mixer 400 (Interscience, St. Nom, France). The dilution was then spread on a Petri dish containing Rogosa agar and incubated at 37 °C in anaerobic jars for 72 h. After 72 h, the colony formed was counted and expressed as colony-forming units per gram (CFU/g) [39].

### 4.14. Microbiological Analysis

Survival of Lactobacillus species in the yoghurt was measured on day 1 and 90 according to the method described by Tontul et al., 2018. In this method, 1 g of yoghurt was diluted with 9 mL of Ringer solution (Merck KGaA, Darmstadt, Germany) and vortexed, and further dilutions were made. The dilutions were then spread in a Petri dish containing Rogosa agar. The colony formed was counted, adjusted to pH 6.5 by adding on 0.1 M of NaOH, and incubated at 37 °C in anaerobic jars for 72 h. For *Streptococcus thermphillus*, the colony was counted by spreading the dilutions in a Petri dish containing M17 agar (Merck KGaA, Darmstadt, Germany), which was incubated aerobically at 37 °C for 48 h [32].

### 4.15. Experimental Animals

All animal-related procedures were conducted under the approval of the Animal Care and Use Committee of the Management and Science University (MSU). The animal ethics committee of MSU granted approval for this study under the animal ethics number of AE-MSU-073. Young adult female Sprague Dawley (SD) rats weighing between 150 g and 200 g (6 weeks old) were used to access acute and subacute toxic effects of *E. tapos* yoghurt for the in vivo study. All rats were acclimatized for 1 week in a temperature-controlled room (22 ± 3 °C) at 12/12 h light/dark cycle. The rats were fed with standard rat pellets containing 306.2 kcal/100 g with 48.8% carbohydrate, 21% protein, and 3% fat, which is equivalent to 12.8 kJ/g, with ad libitum availability. Body weight and 24-hour food intake (kJ) were measured weekly [40].

### 4.16. Acute Toxicity Study

The acute toxicity study was performed according to the protocol described in OECD 425 guidelines. Rats were divided into 2 groups (*n* = 8), where the control group received normal saline (bottle-feeding), while the treatment group received 2000 mg/kg of *E. tapos* yoghurt (force-feeding) for 2 weeks. All rats were observed every 4 h for the next 14 days for any abnormalities in behaviour pattern until euthanasia period. At the end of experiment, all rats were euthanized with CO_2_. Blood was collected in EDTA and plain tube for further biochemical and haematological analysis. Liver, kidney, and heart were excised, weighed, observed macroscopically, and preserved in 10% neutral buffered formalin at room temperature.

### 4.17. Subacute Toxicity Study (Repeated Dose)

Subacute toxicity test was performed according to OECD 407 guidelines on repeated dose of 28-day oral toxicity study in rodents. Rats were divided into 6 groups (*n* = 8), and *E. tapos* yoghurt was orally administered (force-feeding) once daily at a dose of 250, 500, and 1000 mg/kg, while the subacute control (SC) and satellite control (SSC) received a normal saline (bottle-feeding). All rats were observed every 4 h for any abnormalities in behaviour patterns until the euthanasia period. At the end of experiment, all rats were euthanized with CO_2_. Blood was collected in EDTA and plain tube for further biochemical and haematological analysis. Liver, kidney, and heart were excised, weighed, observed macroscopically, and preserved in 10% neutral buffered formalin at room temperature.

### 4.18. Full Blood Count and Plasma Biochemistry

Serum was used to access plasma biochemistry, such as haemoglobin (HB), red blood cell (RBC), red cell distribution width (RDW), packed cell volume (PCV), mean corpuscular volume (MCV), mean corpuscular haemoglobin (MCH), mean corpuscular haemoglobin concentration (MCHC), platelet, and white blood cell (WBC), using automated BC-2800 VET/Mindray machine. Liver profile, such as level creatinine, alkaline phosphatase (ALP), aspartate transaminase (AST), and alanine aminotransferase (ALT), sodium, potassium, chloride, urea, creatinine, and kidney profile test (total protein, albumin, globulin, albumin-globulin ratio), were measured using Alere Cholestech LDX^®^ Analyzer (Alere, UK).

### 4.19. Histopathological Analysis

Tissue processing was performed on all the preserved organs (kidney, heart, and liver). Sectioning was performed to obtain a thin layer of paraffin ribbon with tissue thickness between 4 μm to 7 μm followed by ribbon fishing. Paraffin ribbon was placed in the water bath at a temperature range of 40 °C to 45 °C and was transferred onto a glass slide. All slides were stained using haematoxylin and eosin (H&E) stain. Tissues were then observed under the light microscope, and histological changes were captured [13]. The presence of lesions was scored according to the guideline provided elsewhere [13]. All the scoring was validated by two independent certified pathologists from UPM.

## 5. Conclusions

In the present experiment, newly formulated *E. tapos* yoghurt was analysed for its nutritional content and toxicity in SD female rats. The result of acute and repeated-dose study of up to 2000 mg/kg showed no adverse effects or toxicity towards blood components or serum biochemicals or to the kidney, liver, and heart. These results provide preliminary data for the *E. tapos* yoghurt. Thus, further assessment concerning the genotoxicity, compound toxicity, or subchronic and chronic toxicity study must be done in the future before proceeding with clinical trials, and it is mandatory to further investigate the safety efficacy of the *E. tapos* yoghurt consumption at various ages.

## Figures and Tables

**Table 1:** Nutritional analysis of *E. tapos* yoghurt.

Nutrition Information	Per 100 g
Energy	78 kcal (328 kJ)
Energy/calorie from fat	32 kcal
Fat	3.6 g
Saturated fat	2.8 g
Monounsaturated fat	0.6 g
Polyunsaturated fat	0.1 g
Carbohydrate	8.2 g
Total sugars	3.7 g
Total dietary fibre	1.2 g
Protein	2.6 g
Lactic acid bacteria	1.4 × 10^6^ CFU/g
*Lactobacillus*	1.4 × 10^5^ CFU/g

**Table 2:** Body weight in acute toxicity study. Values expressed as mean ± SEM.

	Body Weight	
	AC	AT 2000
Day 0	206.92 ± 3.62	214.93 ± 3.96
Week 1	272.91 ± 11.04	272.23 ± 7.28
Week 2	286.41 ± 2.80	282.49 ± 8.56

**Table 3:** Food and water intake in acute toxicity study. Values expressed as mean ± SEM.

	AC	AT 2000
Total food consumption (g)	368.90 ± 66.58	374.90 ± 26.97
Calorie intake (KJ)	4721.92 ± 852.16	4798.68 ± 345.22
FER (%)	23.09 ± 4.36	18.24 ± 1.90
Total water intake (mL)	641.67 ± 30.87	653.33 ± 23.33

**Table 4:** Relative organ weight of SD female rats in acute toxicity study. Values expressed as mean ± SEM.

Organs	AC	AT 2000
Relative organ weight (g)		
Liver	8.71 ± 0.69	8.57 ± 0.18
Spleen	0.64 ± 0.05	0.54 ± 0.02
Kidney	1.76 ± 0.08	1.71 ± 0.09
RpWAT	1.58 ± 0.41	1.49 ± 0.39
Visceral fat	1.27 ± 0.25	1.18 ± 0.08
Inguinal fat	2.34 ± 1.23	1.90 ± 0.33
Lung	2.02 ± 0.04	1.70 ± 0.26
Heart	0.83 ± 0.37	1.09 ± 0.06
Percentage per body weight (%)		
Liver	0.03 ± 0.00	0.03 ± 0.00
Spleen	0.00 ± 0.00	0.00 ± 0.00
Kidney	0.54 ± 0.02	0.54 ± 0.03
RpWAT	0.49 ± 0.13	0.47 ± 0.12
Visceral fat	0.39 ± 0.08	0.37 ± 0.03
Inguinal fat	0.72 ± 0.37	0.60 ± 0.10
Lung	0.63 ± 0.02	0.53 ± 0.09
Heart	0.25 ± 0.11	0.34 ± 0.02

**Table 5:** Effect of *E. tapos* yoghurt on haematology parameters in acute toxicity study. Values expressed as mean ± SEM. (*) indicates a significant difference at *p* < 0.05.

Parameters	AC	AT 2000
Hb (g/dL)	13.83 ± 0.78	15.27 ± 0.46
RBC (×10^−12^/L)	7.49 ± 0.33	8.23 ± 0.19
RDW (%)	12.87 ± 0.69	14.00 ± 0.10
PCV (%)	42.67 ± 1.86	45.67 ± 1.20
MCV (fL)	57.00 ± 0.00 *	55.00 ± 0.58 *
MCH (pg)	18.33 ± 0.33	18.67 ± 0.33
MCHC (g/dL)	32.67 ± 0.67	33.67 ± 0.33
WBC (×10^−9^/L)	7.87 ± 2.27	4.17 ± 0.87
Neutrophils (×10^−9^/L)	1.50 ± 0.46	0.90 ± 0.15
Lymphocytes (×10^−9^/L)	5.77 ± 1.78	2.90 ± 0.51
Monocytes (×10^−9^/L)	0.40 ± 0.26	0.23 ± 0.23
Eosinophils (×10^−9^/L)	0.17 ± 0.02	0.12 ± 0.02
Basophils (×10^−9^/L)	0.00 ± 0.00	0.00 ± 0.00
Platelet (×10^−9^/L)	1007.00 ± 90.58	1121.00 ± 245.73

**Table 6:** Effect of *E. tapos* 2000 mg/kg yoghurt on blood biochemistry parameters in acute toxicity study. Values expressed as mean ± SEM. (*) indicates a significant difference at *p* < 0.05.

Parameters	AC	AT 2000
Sodium (mmol/L)	139.33 ± 0.33	139.67 ± 0.33
Potassium (mmol/L)	6.63 ± 0.29	6.93 ± 0.09
Chloride (mmol/L)	107.33 ± 0.33	109.33 ± 1.33
Urea (mmol/L)	11.03 ± 0.19 *	6.60 ± 0.25 *
Creatinine (mmol/L)	29.33 ± 2.33	32.00 ± 3.06
Total Protein (g/L)	65.67 ± 0.67	66.67 ± 0.33
Albumin (g/L)	37.00 ± 1.00 *	41.00 ± 0.58 *
Globulin (g/L)	28.67 ± 0.33 *	25.67 ± 0.33 *
Albumin–Globulin ratio	1.29 ± 0.05 *	1.60 ± 0.04 *
ALP (U/L)	124.00 ± 5.03 *	86.00 ± 1.00 *
AST (U/L)	144.33 ± 12.60 *	73.00 ± 4.51 *
ALT (U/L)	45.33 ± 10.84	23.33 ± 2.85
Gama-Glutamyl Transferase (U/L)	13.00 ± 0.00	13.00 ± 0.00

**Table 7:** Weekly body weight changes for subacute toxicity study. Values are expressed as mean ± SEM. ^1^ The group administered with *E. tapos* yoghurt at various dose daily for 28 days in subacute toxicity study. ^2^ Satellite group for subacute toxicity study, which was given water vehicle or *E. tapos* yoghurt at 1000 mg/kg daily for 28 days followed by no treatment for 14 days. (*) indicates a significant difference at *p* < 0.05.

	Groups for Subacute Toxicity ^1^				Satellite Group for Subacute Toxicity ^2^	
Body Weight (g)	SC	ST 250	ST 500	ST 1000	SSC	SST 1000
Day 0	171.24 ± 4.86	171.78 ± 4.44	171.93 ± 5.43	168.59 ± 6.34	155.69 ± 10.08	160.40 ± 10.32
Week 1	172.40 ± 6.83 *	199.70 ± 7.43 *	194.93 ± 6.73	187.76 ± 4.37	155.96 ± 10.91	191.55 ± 3.81
Week 2	203.43 ± 6.52	218.51 ± 9.40	214.76 ± 6.49	209.53 ± 4.18	199.34 ± 8.21	214.63 ± 3.22
Week 3	218.64 ± 7.70	231.42 ± 10.01	235.07 ± 6.43	227.32 ± 4.87	215.32 ± 8.81	230.84 ± 5.19
Week 4	242.93 ± 3.39	251.75 ± 11.01	248.42 ± 6.43	242.31 ± 6.94	237.62 ± 6.02	249.60 ± 5.20
Week 5	-	-	-	-	247.51 ± 5.49	260.06 ± 6.47
Week 6	-	-	-	-	260.99 ± 5.45	274.95 ± 7.39

## Data Availability

Not applicable.
