# Correction: Naomi et al. *E. tapos* Yoghurt—A View from Nutritional Composition and Toxicological Evaluation. *Foods* 2022, *11*, 1903

**DOI:** 10.3390/foods13071046

**Published:** 2024-03-29

**Authors:** Ruth Naomi, Rusydatul Nabila Mahmad Rusli, Santhra Segaran Balan, Fezah Othman, Azmiza Syawani Jasni, Siti Hadizah Jumidil, Hasnah Bahari, Muhammad Dain Yazid

**Affiliations:** 1 Department of Human Anatomy, Faculty of Medicine and Health Sciences, Universiti Putra Malaysia, Serdang 43400, Malaysia; ruthmanuel2104@gmail.com (R.N.); rusydatulnabila17@gmail.com (R.N.M.R.); santhra@msu.edu.my (S.S.B.); hadizah_jumidil@upm.edu.my (S.H.J.); 2 Faculty of Health and Life Sciences, Management and Science University, Shah Alam 40100, Malaysia; 3 Department of Biomedical Sciences, Faculty of Medicine and Health Sciences, Universiti Putra Malaysia, Serdang 43400, Malaysia; fezah@upm.edu.my; 4 Department of Medical Microbiology and Parasitology, Faculty of Medicine and Health Sciences, Universiti Putra Malaysia, Serdang 43400, Malaysia; azmiza@upm.edu.my; 5 Centre for Tissue Engineering and Regenerative Medicine, Faculty of Medicine, Universiti Kebangsaan Malaysia, Kuala Lumpur 56000, Malaysia

## Text Correction

(1) In the original publication [1], there was no explanation on 0.00 value in Table 4. The correct value for the relative organ weight of the spleen is 0.001, as accurately reflected in the table below. An unintended formatting discrepancy occurred during the preparation of the manuscript, where three decimal points were included, rendering the value as 0.00. To maintain consistency with other tables in the manuscript, we will correct the formatting to present the value as 0.00 in accordance with the established convention. The relevant explanations have also been added after the first paragraph of Section 2.2.4 of the text.




**Organ Weight**

**Relative Organ Weight (%)**

Mean0.640.001983358ACM (Acute control)SEM0.050.000127909






**Organ Weight**

**Relative Organ Weight (%)**

Mean0.540.001694742AT2000 (Acute Tapos 2000 mg/kg)SEM0.020.000086809

(2) In the original publication [1], the first sentence of Section 4.16. The text incorrectly cites OECD 407 instead of the correct OECD 425 guidelines under which the acute toxicity study was conducted. We wish to clarify that this error does not alter the protocol we followed nor the outcomes of the study. Both OECD 407 and OECD 425 guidelines pertain to toxicity studies, but our methodology and results remain valid under the correctly applied OECD 425 framework. This mistake was an oversight in the manuscript’s drafting and not in the execution of the research itself.

## Error in Figure

In the original publication [1], there was a mistake in Figure 2 as published. There are two identical micrographs in Figure 2. The figures are indeed correct, and the structures depicted appear normal without any abnormalities. However, in an effort to ensure absolute clarity for the readers, the section has been revised and images were replaced with new ones from the respective experimental group.

The corrected Figure 2 appears below.

**Figure 2:**
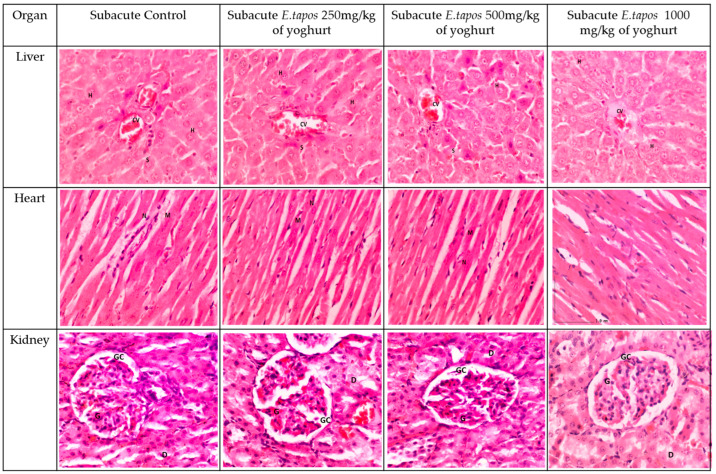
The histology section for subacute studies. The liver section shows standard strands of hepatocytes (H), sinusoids (S), and central vein (CV). It shows 0% of biopsied hepatocytes affected. The liver grading scores were documented as 0 due to absence of steatosis, lobular inflammation, and hepatocyte ballooning. The heart tissue showing normal heart architecture and no inflammation was observed. Myocardium (M) and nucleus (N). Kidney histology showed no lesion or tubular dilation. Renal corpuscle appeared normal. The kidney shows normal architecture of glomerulus (G), glomerulus capsule (GC), distal convoluted tubule (D). No abnormalities observed in the liver, kidney, or heart in subacute toxicity groups.

The authors state that the scientific conclusions are unaffected. This correction was approved by the Academic Editor. The original publication has also been updated.

## References

[1] Naomi R., Rusli R.N.M., Balan S.S., Othman F., Jasni A.S., Jumidil S.H., Bahari H., Yazid M.D. (2022). *E. tapos* Yoghurt—A View from Nutritional Composition and Toxicological Evaluation. Foods.

